# Multiphysics and Multiscale Modeling of Coupled Transport of Chloride Ions in Concrete

**DOI:** 10.3390/ma14040885

**Published:** 2021-02-13

**Authors:** Amit Jain, Bora Gencturk

**Affiliations:** Sonny Astani Department of Civil and Environmental Engineering, University of Southern California, Los Angeles, CA 90089, USA; amitjn7042@gmail.com

**Keywords:** chloride diffusion, modeling, concrete, microstructure, corrosion, chloride binding, composite theory

## Abstract

Chloride ions (Cl^−^)-induced corrosion is one of the main degradation mechanisms in reinforced concrete (RC) structures. In most situations, the degradation initiates with the transport of Cl^−^ from the surface of the concrete towards the reinforcing steel. The accumulation of Cl^−^ at the steel-concrete interface could initiate reinforcement corrosion once a threshold Cl^−^ concentration is achieved. An accurate numerical model of the Cl^−^ transport in concrete is required to predict the corrosion initiation in RC structures. However, existing numerical models lack a representation of the heterogenous concrete microstructure resulting from the varying environmental conditions and the indirect effect of time dependent temperature and relative humidity (RH) on the water adsorption and Cl^−^ binding isotherms. In this study, a numerical model is developed to study the coupled transport of Cl^−^ with heat, RH and oxygen (O_2_) into the concrete. The modeling of the concrete microstructure is performed using the Virtual Cement and Concrete Testing Laboratory (VCCTL) code developed by the U.S. National Institute of Standards and Technology (NIST). The concept of equivalent maturation time is utilized to eliminate the limitation of simulating concrete microstructure using VCCTL in specific environmental conditions such as adiabatic. Thus, a time-dependent concrete microstructure, which depends on the hydration reactions coupled with the temperature and RH of the environment, is achieved to study the Cl^−^ transport. Additionally, Cl^−^ binding isotherms, which are a function of the pH of the concrete pore solution, Cl^−^ concentration, and weight fraction of mono-sulfate aluminate (AFm) and calcium-silicate-hydrate (C-S-H), obtained from an experimental study by the same authors are utilized to account for the Cl^−^ binding of cement hydration products. The temperature dependent RH diffusion was considered to account for the transport of Cl^−^ with moisture transport. The temperature and RH diffusion in the concrete domain, composite theory, and Cl^−^ binding and water adsorption isotherms are used in combination, to estimate the ensuing Cl^−^ diffusion field within the concrete. The coupled transport process of heat, RH, Cl^−^, and O_2_ is implemented in the Multiphysics Object-Oriented Simulation Environment (MOOSE) developed by the U.S. Idaho National Laboratory (INL). The model was verified and validated using data from multiple experimental studies with different concrete mixture proportions, curing durations, and environmental conditions. Additionally, a sensitivity analysis was performed to identify that the water-to-cement (w/c) ratio, the exposure duration, the boundary conditions: temperature, RH, surface Cl^−^ concentration, Cl^−^ diffusion coefficient in the capillary water, and the critical RH are the important parameters that govern the Cl^−^ transport in RC structures. In a case study, the capabilities of the developed numerical model are demonstrated by studying the complex 2D diffusion of Cl^−^ in a RC beam located in two different climatic regions: warm and humid weather in Galveston, Texas, and cold and dry weather in North Minnesota, Minnesota, subjected to time varying temperature, RH, and surface Cl^−^ concentrations.

## 1. Introduction

The transport of chloride ions (Cl^−^) from the concrete surface is the most influential aspect of corrosion-induced deterioration in reinforced concrete (RC) structures. In marine environments, airborne Cl^−^ or Cl^−^ from seawater is carried into concrete over time and this process is usually exacerbated by the wetting and drying cycles. Similarly, in cold regions, the concrete surface is subjected to deicing salts that penetrate into the concrete through various mechanisms including sorption and diffusion. Cl^−^ may also be present in the concrete pore structure when Cl^−^ bearing salts (such as calcium chloride: CaCl_2_) are used in the fresh concrete to accelerate the hydration process and achieve a higher early strength. The presence of Cl^−^ beyond a threshold value at the steel-concrete interface starts the corrosion-induced degradation process. Therefore, a study of the transport of Cl^−^ is essential to estimate the time for corrosion initiation as well as for corrosion propagation in service life estimation of RC structures.

Transport of Cl^−^ in concrete broadly depends on the boundary conditions at the concrete surfaces (e.g., temperature, relative humidity (RH), the surface Cl^−^ concentration, C_s_) and the properties of the concrete domain (e.g., porosity, tortuosity, Cl^−^ binding capacity, pH, presence of other ions in the pore solution, and pre-existing micro-cracks). The temperature of the concrete surface changes the temperature profile in the concrete domain, which in turn affects the mobility of Cl^−^ in concrete [1]. Similar to heat diffusion, water flux from the concrete surface to concrete domain also contributes to the transport of Cl^−^ [2]. At the same time, temperature and RH affect the maturity of concrete [3]. Temporal and spatial distributions of temperature and RH in the concrete domain result in a time varying heterogenous concrete microstructure due to nonuniform concrete maturity. Surface Cl^−^ concentration is another important parameter that affects the Cl^−^ transport in concrete. Cl^−^ from the environment progressively builds up on the concrete surface. The accumulation of the surface Cl^−^ depends on the duration and the type of exposure [4], the concrete mixture properties such as the water-to-cement (w/c) ratio [5], the presence and the proportion of supplementary cementitious materials (SCMs) [5], the location of the structure [4,5,6], and the orientation of the concrete surface with respect to the exposure. The surface Cl^−^ concentration was found to follow a linear relationship with duration of exposure in log scale as observed by Song et al. [5] and Costa and Appleton [4]. Song et al. [5] also reported that the surface Cl^−^ concentration decreases hyperbolically as the distance from the coast increases. Vu et al. [7] presented, based on measurements from 321 concrete bridge decks in a coastal/marine environment in the United States, that the mean and coefficient of variation of lognormal distribution of airborne Cl^−^ are 3.5 kg/m^3^ and 0.5 kg/m^3^, respectively. The data were obtained by assuming that the surface Cl^−^ concentration was in equilibrium with airborne Cl^−^ concentration and it is constant over time. The surface Cl^−^ concentration was estimated by curve fitting to Fick’s law, the observed Cl^−^ concentration profile in the concrete domain. It was noted that there is a large variation in the data due to the varying amounts of deicing salts applied on the bridge decks, the efficiency of drainage, and the quality of expansion joints.

Apart from the influence of the boundary conditions, Cl^−^ transport is also affected from interactions with the components of hydrated cement paste as the Cl^−^ transport takes place through the concrete pore solution (CPS). A part of the Cl^−^ binds to the surface of the components of hydrated cement paste such as mono-sulfate aluminate (AFm) and calcium-silicate-hydrate (C-S-H). Thus, the total Cl^−^ is present in two forms in the concrete microstructure: free Cl^−^ in the CPS and bound Cl^−^ within or on the concrete solids. The bound Cl^−^ is usually not released to the CPS and the flow of Cl^−^ becomes restricted [8]. Due to Cl^−^ binding of concrete, pore size distribution and tortuosity, Shi et al. [9] observed that the free Cl^−^ diffusivity reduces to 1/3 of the total Cl^−^ diffusivity in the concrete and Li et al. [10] found that the Cl^−^ diffusion in concrete is 3–4 orders of magnitude lower than that in pure water. Since the concrete maturates differently at different temperatures and RHs, a single value for the Cl^−^ diffusion coefficient, as reported in various experimental studies [11,12,13,14], cannot represent the whole concrete domain. Researchers [2,15,16,17,18,19] used different methodologies to account for the effect of environmental conditions (temperature and RH) and properties of concrete microstructure to study Cl^−^ transport in the concrete domain; however, the heterogeneity of the concrete microstructure is rarely considered. Saetta el al. [2] included the effects of temperature, moisture flux, and concrete maturity in the estimation of the Cl^−^ diffusion coefficient; however, the whole concrete domain was assumed to achieve the same maturity at a given time. Nielsen and Geiker [16] and Yang and Wang [18] used the Power’s model [20] to obtain the weight fractions of different phases of hydrated cement-paste system (which includes the anhydrous cement, capillary (free) water, gel solids, and gel water). These properties were then used in a composite theory to estimate the Cl^−^ diffusion coefficient as a unique value for the whole concrete domain. Yang and Wang [18] developed a model that estimates the effective ionic Cl^−^ diffusion coefficient in the capillary pores by accounting for the electro-kinetic effect (using the Nernst–Planck equation) and the electric field from the zeta potential of the negatively charged pore walls (using the Poisson’s equation). Lattice Boltzmann method was utilized in the modeling to study the diffusion and evolution of the electric field potential in the concrete domain. Li et al. [10] emphasized the importance of pore size and its distribution. The concrete microstructure was divided into solid and liquid phases. The liquid phase was further divided into capillary pores (pore sizes ranging from 10 nm to 10 μm) and gel pores (pore sizes ranging from 0.5 nm to 10 nm) to apply the Fick’s law of diffusion in both pores while considering the absorption of Cl^−^ in the individual pore systems and the mass transfer between capillary and gel pores. The effect of an ionic exchange between the pores, binding of ions on the solids, and the boundary layer of exposed surface in the transport of Cl^−^ in concrete were considered. However, the model by Li et al. [10] considered a fully saturated concrete microstructure, which is not representative of certain environmental conditions. Ann and Hong [19] considered concrete pores to have a Rayleigh–Ritz distribution based on experimental results from mercury intrinsic porosimetry (MIP) measurements for pore sizes ranging from 0.01 μm to the maximum size measured for the specific concrete samples. Concrete porosity and the Cl^−^ diffusion coefficient were estimated from the pore the size distribution and tortuosity. The model also considered the Cl^−^ binding to estimate the Cl^−^ concentration in the concrete domain.

In summary, existing numerical models [2,15,16,17,18,19] do not consider the heterogeneity in the concrete microstructure while considering the simultaneous impact of temperature and RH, water adsorption and Cl^−^ binding isotherms, and the composite theory on the transport of Cl^−^. These shortcomings in previous studies result in the same material diffusivity parameters or concrete maturity for the whole concrete domain which is not the case in reality. The cement hydration in most studies was studied based on the Power’s method [20], which does not account for the instantaneous temperature and RH in the concrete domain [16,18]. Additionally, most of the modeling approaches developed in literature were limited to one dimensional diffusion simulated under fixed concrete properties. To address these shortcomings, a model is developed as described in the following that accounts for the temporal and spatial variations in concrete microstructural properties coupled with time-varying environmental conditions of temperature, RH, and surface Cl^−^ concentration.

## 2. Methodology

### 2.1. Overview

**Figure 1:**
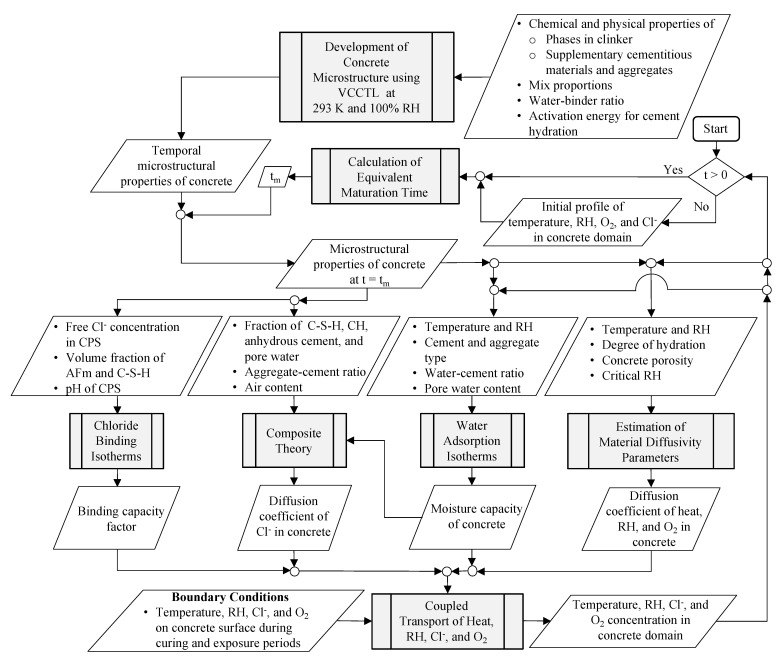
Flowchart of the developed model.

A flowchart of the modeling approach developed in this paper is provided in Figure 1. At first, initial profiles of temperature, RH, Cl^−^, and oxygen (O_2_) in the concrete domain are determined to start the transport analysis. Subsequently, the equivalent maturation time, t_m_, is obtained based on [21] and given temperature and RH conditions during the curing and exposure periods. The Virtual Cement and Concrete Testing Laboratory (VCCTL) [22,23] is utilized to develop the concrete microstructure for the given mixture proportions, chemical and physical properties of the cement, aggregates, and admixtures, and the hydration characteristics of the cement. Specifically, the following properties of the hydrated cement paste are obtained from VCCTL: weight fractions of components (AFm, calcium hydroxide (CH: CaOH_2_), and C-S-H), pH of the CPS, pore water content, and the weight fraction of the anhydrous cement. Since VCCTL [22,23] is limited to hydration of cement only in certain environmental conditions, i.e., isothermal, semi-adiabatic and adiabatic in saturated or sealed conditions, hydration of concrete is carried out isothermally at 293 K in saturated conditions. Then, the output from VCCTL [22,23] is used at previously calculated equivalent maturation times to obtain the spatially and time varying concrete microstructure properties at each location in the concrete domain. Further, the water adsorption isotherm from [24] is considered to predict the moisture capacity of concrete, which is dependent on the type of cement and aggregate, water-to-cement (w/c) ratio, fraction of pore water, temperature, and RH. In parallel, the free Cl^−^ concentration, pH of the CPS, and the fractions AFm and C-S-H are input into the Cl^−^ binding isotherms [25] to estimate the Cl^−^ binding capacity of the components of hydrated cement paste (i.e., C-S-H and AFm) at varying pH levels of the CPS. The composite theory is utilized to estimate the Cl^−^ diffusion coefficient of concrete by accounting for diffusion through C-S-H, CH, anhydrous cement, pore water, aggregates, and air voids. The diffusion coefficients for heat and RH are also estimated for known degree of hydration of cement, temperature, and RH. Since the chloride-induced degradation starts with the corrosion reactions at the steel-concrete interface, O_2_ transport is also included in the modeling of the coupled transport of heat, RH, and Cl^−^. Thus, the material diffusivity parameters, binding capacity factor, and the moisture capacity are utilized to solve the coupled transport of temperature, RH, Cl^−^, and O_2_. The modeling framework is developed and implemented in the Multiphysics Object-Oriented Simulation Environment (MOOSE) [26] developed by the U.S. Idaho National Laboratory (INL). Verification, sensitivity and validation studies are performed. Finally, to demonstrate the full capabilities of the model, a bridge beam located in the warm and humid environmental conditions of Galveston, Texas, and cold and dry environmental conditions of North Minnesota, Minnesota, is analyzed as a case study and the results are compared.

### 2.2. Governing Equations

Cl^−^, water, and O_2_ from the environment diffuse into the concrete, and this diffusion can be represented in terms of a flux. The flux, **J_c_**, of a species through unit area of concrete in unit time is expressed using Fick’s first law as

**J_c_** = −**D_c_** · **∇** (C) (1)

where, C and **D_c_** are, respectively, the concentration and the diffusion coefficient of the species in concrete and (·) is a scalar product of two vectors. The rate of change of species concentration at any point in concrete is equal to the net flux; hence, Fick’s second law expresses mass conservation of species as (given that no source/sink inside concrete domain exists)

(2) $$\frac{\partial C}{\partial t}=-\nabla \cdot J_{c}$$

where t is time and **∇** = $\left(\frac{\partial }{\partial x},\frac{\partial }{\partial y},\frac{\partial }{\partial z}\right)$. From Equations (1) and (2), the governing equation for the diffusion of species in concrete is obtained as

(3) $$\frac{\partial C}{\partial t}=\nabla \cdot (D_{c}\cdot \nabla \;(C))$$

**Figure 2:**
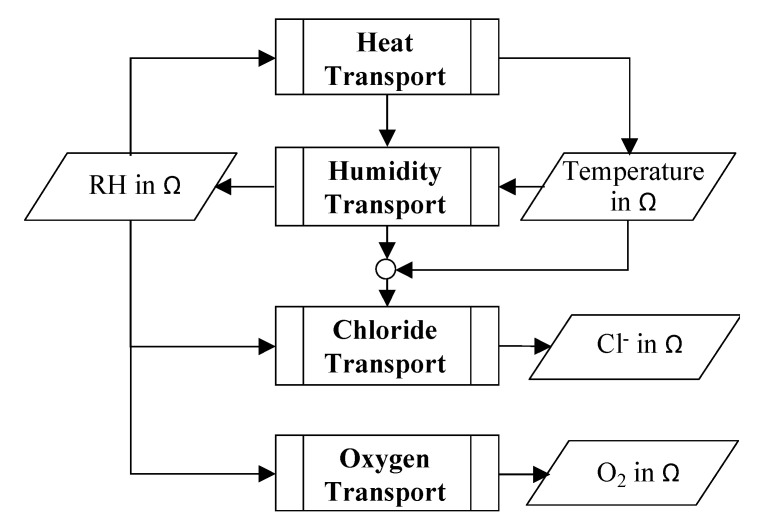
Coupling of heat, RH, Cl^−^, and O_2_ transport in concrete (Ω represents the concrete domain).

Equation (3) also expresses the rate of change of species concentration in concrete. Since heat diffusion is faster than the diffusion of RH, Cl^−^, and O_2_, the heat diffusion is tackled first (as shown in Figure 2) to determine the temperature profile in the domain according to

(4) $$\frac{\partial T}{\partial t}=\nabla \cdot (D_{T}\cdot \nabla \;(T))$$

where **D_T_** is the heat diffusion coefficient and T is the temperature. Since the temperature affects the mobility of species and their diffusion processes, the temperature profile is used to estimate the modeling parameters of RH and Cl^−^ diffusion as indicated in Figure 2. The RH profile in the concrete domain is obtained according to [2,15,16,27].

(5) $$\frac{\partial \mathrm{RH}}{\partial t}=\nabla \cdot (D_{\mathrm{RH}}(\mathrm{RH},\;T)\cdot \nabla (\mathrm{RH}))+\frac{{\mathrm{dRH}}_{s}}{\mathrm{dt}}+K\times \frac{\mathrm{dT}}{\mathrm{dt}}$$

where **D_RH_** is the RH diffusion coefficient, ${\mathrm{RH}}_{s}=\frac{0.85\times t_{m}+15}{t_{m}+15}$ is the RH at self-desiccation of a sealed sample [21], $\frac{{\mathrm{dRH}}_{s}}{\mathrm{dt}}=\frac{-2.25}{{\left(t_{m}+15\;\right)}^{2}}\times \frac{{\mathrm{dt}}_{m}}{\mathrm{dt}}$, t_m_ is equivalent maturation time (which is defined later in Section 2.3), $K\frac{\mathrm{dT}}{\mathrm{dt}}$ is the coupling term with heat diffusion, and K is the heat diffusion coupling coefficient (=0.0135 × RH × (1 − RH)/(1.25 − RH)) [3]. Similarly, the transport of O_2_ and Cl^−^ is also governed by Fick’s law according to

(6) $$\frac{\partial O_{2}}{\partial t}=\nabla \cdot (D_{O2}\cdot \nabla \;[O_{2}])$$

(7) $$\frac{\partial C_{t}}{\partial t}=\nabla \cdot (D_{i}\cdot \nabla \;(C_{f}))$$

where [O_2_] is the O_2_ concentration, C_t_ is the total Cl^−^ concentration, $C_{f}$ is the free Cl^−^ concentration, **D_O2_** is the O_2_ diffusion coefficient in concrete, and $D_{i}$ is the intrinsic Cl^−^ diffusion coefficient. A relationship between the free, C_f_, bound, C_b_, and the total Cl^−^ concentration, C_t_, in the concrete is expressed as

C_t_ = C_b_ + w_e_ × C_f_ (8)

where w_e_ is the evaporable water content in the concrete. By differentiating Equation (8) with respect to time, a relationship between the rate of change of total Cl^−^ is obtained in terms of the rate of change of free Cl^−^, bound Cl^−^, and the evaporable water content as

(9) $$\frac{\partial C_{t}}{\partial t}=\frac{\partial C_{b}}{\partial t}+w_{e}\times \frac{\partial C_{f}}{\partial t}+C_{f}\times \frac{\partial w_{e}}{\partial \mathrm{RH}}\times \frac{\partial \mathrm{RH}}{\partial t}$$

Using Equations (7) and (9), the rate of change of free Cl^−^ is expressed in terms of free Cl^−^, evaporable water content, and Cl^−^ binding capacity factor, $\xi =\left(\frac{\partial C_{b}}{\partial C_{f}}+w_{e}\right)$, according to

(10) $$\frac{\partial C_{f}}{\partial t}=\frac{1}{\xi }\times \nabla \cdot \left(D_{i}\cdot \nabla \left(C_{f}\right)\right)-\frac{C_{f}}{\xi }\times \frac{\partial w_{e}}{\partial \mathrm{RH}}\times \frac{\partial \mathrm{RH}}{\partial t}$$

Thus, in this study, the heat transport is considered to be independent of the transport of RH, Cl^−^, and O_2_ (as indicated in Equation (4) and Figure 2). This assumption is because the heat diffusion coefficient is about 4–5 orders of magnitude higher than the Cl^−^ and O_2_ diffusion coefficients. However, the diffusion coefficient for heat transfer is considered to be dependent (as described in the next section) on the degree of hydration of concrete, which further depends on RH; therefore, heat transfer in Figure 2 is shown to be dependent on RH in the concrete domain although Equation (4) does not directly reflect this dependence. The diffusion coefficient for RH transport is one order of magnitude higher than the Cl^−^ and O_2_ diffusion coefficients; therefore, the RH transport is considered to be only dependent on the temperature as indicated in Equation (5) and shown in Figure 2. Due to the higher rate of heat transfer, the temperature profile in concrete is assumed to achieve a steady state while studying transport of Cl^−^ in concrete. Thus, the Cl^−^ transport in concrete is assumed to be dependent on the temperature profile in concrete domain instead of the temporal change in the temperature profile. Moreover, the Cl^−^ transport is considered to be dependent on RH and water flux in concrete as indicated in Equation (10). Similarly, the transport of O_2_ is dependent on the temperature and RH profile in the concrete domain. Backward and interdependency of Cl^−^ and O_2_ concentration on the heat and RH transport are not considered in this study. In other words, the Cl^−^ and O_2_ concentrations do not affect the heat or RH diffusion.

### 2.3. Modeling of Concrete Microstructural Development

The concrete microstructure evolves over time as a result of ongoing hydration reactions; therefore, this change in microstructural properties of concrete should be considered for accurate modeling of the corrosion reactions. Among the two most commonly used software to simulate the hydration of cement, VCCTL [22,23] is selected here over HYMOSTRUC3D [28] for its simplicity in representation of the particle size distribution and chemical compositions of the anhydrous cement phases. A flow chart to obtain concrete microstructural properties is shown in Figure 1. The phase fractions of clinker components (tri-calcium silicate (C_3_S: 3CaO·SiO_2_), di-calcium silicate (C_2_S: 2CaO·SiO_2_), tri-calcium aluminate (C_3_A: 3CaO·Al_2_O_3_), and tetra-calcium aluminoferrite (C_4_AF: 4CaO·Al_2_O_3_·Fe_2_O_3_)); the particle size distributions of these components; the alkali (K_2_O and Na_2_O) content of cement; the mass fraction of sulfates (gypsum (CaSO_4_·2H_2_O), basanite (CaSO_4_·0.5H_2_O), and calcium sulfate anhydrate (CaSO_4_)); the density, Ca/Si molar ratio, and molar volume of slag and slag gel hydration products; the phase fraction of fly ash (aluminosilicate glass, calcium aluminum disilicate, C_3_A, calcium chloride (CaCl_2_), Si, and CaSO_4_); the density and particle size distribution of fillers (α-quartz (SiO_2_), corundum (Al_2_O_3_), and periclase (MgO)); and the specific gravity, linear elastic moduli, and particle shape of fine and coarse aggregates are used in VCCTL [22,23] to obtain the weight fractions of hydrated cement components (AFm, ettringite (AFt: Ca_6_Al_2_(SO_4_)_3_(OH)_12_·26H_2_O), ferric hydroxide (Fe(OH)_3_), hydrogarnet (3CaO·Al_2_O_3_·6H_2_O), C-S-H, CH, Friedel’s salt (3CaO·Al_2_O_3_·CaCl_2_·10H_2_O), calcium carbonate (CaCO_3_), stratlingite (Ca_4_Al_2_(OH)_12_ [AlSi(OH)_8_]_2_·2H_2_O), calcium monocarboaluminate (3CaO·Al_2_O_3_·CaCO_3_·10.7H_2_O), brucite (Mg(OH)_2_)); the porosity, chemical shrinkage, non-evaporable water content, degree of hydration, heat of hydration, and the temperature of the cement paste system; the conductivity of species through a phase relative to the conductivity through the CPS; and the concentrations of Na^+^, K^+^, Ca^2+^, ${\mathrm{SO}}_{4}^{2-}$, and OH^−^ in the CPS. Results from VCCTL [22,23] were experimentally validated for cements having different particle size distribution and composition of C_2_S, C_3_S, C_3_A, and C_4_AF in [29]. Since VCCTL [22,23] can simulate the hydration of the components of cement only in isothermal, semi-adiabatic and adiabatic in saturated or sealed conditions, the concrete microstructure is developed in this study in isothermal (at reference temperature, T^ref^ = 293 K) and saturated conditions, i.e., RH^ref^ = 100%. For this reason, the concept of equivalent maturation time developed by Bazant [21] is used to account for the real environmental conditions that exist during the curing and exposure of RC structures. The equivalent maturation time is the time required for the consumption of water during the hydration reactions at a given temperature and RH in terms of the time required for the consumption of water during the hydration reactions at 293 K and 100% RH, which are the reference temperature and RH. The equivalent maturation times, for given temperature and RH, are calculated here for curing, $t_{m}^{\mathrm{Cu}}$, and exposure periods, $t_{m}^{\exp}$, in terms of the reference temperature and RH, according to

(11) $$t_{m}=t_{m}^{\mathrm{cu}}+t_{m}^{\exp}$$

(12) $$t_{m}^{X}=\int \beta_{T}^{X}\times \beta_{\mathrm{RH}}^{X}\;\mathrm{dt}\;\forall \;X\;\in \;\{\mathrm{cu},\;\exp\}$$

(13) $$\beta_{T}^{X}=\exp\left(\frac{{\Delta U}_{\mathrm{hyd}}}{R}\times \left(\frac{1}{T^{\mathrm{ref}}}-\frac{1}{T^{X}}\right)\right)\;\forall \;X\;\in \;\{\mathrm{cu},\;\exp\}$$

(14) $$\beta_{\mathrm{RH}}^{X}=(1+(3.5\times (1-{\mathrm{RH}}^{\mathrm{ref}}){)}^{4})/(1+(3.5\times (1-{\mathrm{RH}}^{X}){)}^{4})\;\forall \;X\;\in \;\{\mathrm{cu},\;\exp\}$$

where T^ref^ and RH^ref^ are the reference temperature and RH as mentioned earlier, T^X^ and RH^X^ are temperature and RH profiles during curing (X = cu) and exposure (X = exp) periods, and ${\Delta U}_{\mathrm{hyd}}$ is the activation energy for cement hydration (=22.4 kJ/mol [2]). In the modeling, temporal microstructural properties of concrete at the reference temperature and RH for the entire exposure period expected in the simulations are imported in MOOSE [26] from VCCTL [22,23] at the beginning of the initial time step. Subsequently, the equivalent maturation time is calculated at all nodes in the concrete domain for the corresponding temperature and RH values (which are obtained from the coupled transport equations at the end of the previous time step). The imported microstructural properties from VCCTL, at t = t_m_, are assigned to each node in the concrete domain. Thus, the microstructural properties at the equivalent maturation time are obtained for each node in the concrete domain and for each time step to represent the heterogeneity in the concrete both spatially and temporally.

### 2.4. Water Adsorption and Chloride Binding Isotherms

The free Cl^−^ content in the CPS depends on the pore water content. The pore water in the concrete is present in two forms: adsorbed and free water. Xi et al. [24] reported that the moisture capacity of the concrete depends on the concrete mixture proportions (w/c ratio, curing time, and type of cement and aggregates) and the exposure conditions (temperature and RH during curing and exposure periods). The moisture capacity is represented by the slope of the equilibrium water adsorption isotherm. A water adsorption isotherm for cement paste, as obtained by Powers and Brownyard [20] and predicted by Xi [30] using the Brunauer–Skalny–Bodor (BSB) model, a generalization of the Brunauer–Emmett–Teller (BET) model, describes the effect of different concrete mixture proportions, temperature, and RH during curing and exposure [24]. The BSB model estimates the adsorbed water content on the cement paste, w_ad|CP_, and aggregate, w_ad|AG_, as [24]

(15) $$w_{\mathrm{ad}|\mathrm{CP}}=\frac{C\times k_{\mathrm{CP}}\times V_{m|\mathrm{CP}}\times \mathrm{RH}}{\left(1-k_{\mathrm{CP}}\times \mathrm{RH}\right)\times \left(1+\left(C-1\right)\times k_{\mathrm{CP}}\times \mathrm{RH}\right)}$$

(16) $$w_{\mathrm{ad}|\mathrm{AG}}=\frac{C\times k_{\mathrm{AG}}\times V_{m|\mathrm{AG}}\times \mathrm{RH}}{\left(1-k_{\mathrm{AG}}\times \mathrm{RH}\right)\times \left(1+\left(C-1\right)\times k_{\mathrm{AG}}\times \mathrm{RH}\right)}$$

where $C=\exp\left(\frac{855}{T}\right)$, $V_{m|\mathrm{CP}}$ (=0.9, 1.1, 0.85, and 0.6 for Type I, II, III, and IV cement, respectively) and $V_{m|\mathrm{AG}}$ (=0.025 and 0.075 for lightweight and dense aggregate, respectively) are the monolayer moisture adsorption capacities of the cement paste and the aggregate from the BET model, respectively; k_CP_ $\left(=\frac{\left(1-\frac{1}{n_{\mathrm{CP}}}\right)\times C-1}{\left(C-1\right)}<1\right)$ and k_AG_ $\left(=\frac{\left(1-\frac{1}{n_{\mathrm{AG}}}\right)\times C-1}{\left(C-1\right)}<1\right)$ are constants to account for the number of adsorbed layers of water on cement paste and aggregate, and n_CP_ (=1.0, 1.15, 1.5, and 1.1 for Type I, II, III, and IV cement, respectively) and n_AG_ (=1.85 and 1.25 for lightweight and dense aggregate, respectively) are the number of adsorbed layer of water on the cement paste and aggregate, respectively. The water adsorption isotherm, in the BSB model, is assumed to depend on the type of cement and the type of aggregates, the temperature, the w/c ratio, and the age of concrete. In [24], the moisture capacity of cement paste and aggregate are expressed as

(17) $$\frac{\partial w_{\mathrm{ad}|\mathrm{CP}}}{\partial \mathrm{RH}}=\frac{C\times k_{\mathrm{CP}}\times V_{m|\mathrm{CP}}+W_{\mathrm{ad}|\mathrm{CP}}\times k_{\mathrm{CP}}\times \left(1+\left(C-1\right)\times k_{\mathrm{CP}}\times \mathrm{RH}\right)-W_{\mathrm{ad}|\mathrm{CP}}\times k_{\mathrm{CP}}\times \left(1-k_{\mathrm{CP}}\times \mathrm{RH}\right)\times \left(C-1\right)}{\left(1-k_{\mathrm{CP}}\times \mathrm{RH}\right)\times \left(1+\left(C-1\right)\times k_{\mathrm{CP}}\times \mathrm{RH}\right)}$$

(18) $$\frac{\partial w_{\mathrm{ad}|\mathrm{AG}}}{\partial \mathrm{RH}}=\frac{C\times k_{\mathrm{AG}}\times V_{m|\mathrm{AG}}+W_{\mathrm{ad}|\mathrm{AG}}\times k_{\mathrm{AG}}\times \left(1+\left(C-1\right)\times k_{\mathrm{AG}}\times \mathrm{RH}\right)-W_{\mathrm{ad}|\mathrm{AG}}\times k_{\mathrm{AG}}\times \left(1-k_{\mathrm{AG}}\times \mathrm{RH}\right)\times \left(C-1\right)}{\left(1-k_{\mathrm{AG}}\times \mathrm{RH}\right)\times \left(1+\left(C-1\right)\times k_{\mathrm{AG}}\times \mathrm{RH}\right)}$$

The water in the CPS may not be completely available for the transport of Cl^−^ in the concrete microstructure. The total water present in the concrete, w, is in two forms as evaporable water, w_e_, and non-evaporable water, w_n_, when the presence of water vapor is neglected. The relation between the total water, the evaporable water, and the non-evaporable water is given by

w = w_e_ + w_n_ (19)

A part of the evaporable water content gets physically adsorbed on to the concrete surface and is denoted by w_ad_ while the remaining part of the evaporable water content remains in the CPS as capillary water, w_cw_. Thus, a relationship between w_ad_ (which constitutes adsorbed water content on the cement paste, w_ad|CP_, and that on the aggregate, w_ad|AG_ as in Equation (15) above) and w_cw_ is obtained as

w_e_ = w_ad|CP_ + w_ad|HCP_ + w_cw_ (20)

Since the water adsorption depends on both the temperature and the RH [24], the water adsorption isotherm from Xi [24] is used to estimate the adsorbed water content on the surface of cement paste and aggregate; and thus, the capillary water in the CPS. The adsorbed water and the moisture capacity are obtained from Equations (15)–(18). Equation (20) is used in Equation (10) to consider the effect of moisture capacity of concrete microstructure on the transport of Cl^−^.

As described earlier, the Cl^−^ transport through the concrete microstructure towards the steel reinforcement. During the transport, a part of Cl^−^ binds to concrete either physically (on the surface of C-S-H) or chemically (through reactions with AFm). The equilibrium relationships between free and bound Cl^−^ were obtained by various researchers. Recently, Jain et al. [25] studied the Cl^−^ binding isotherms of AFm and C-S-H in synthesized CPS having varying pH values to account for the effect of pH and the presence of other ions such as Ca^2+^, Na^+^, K^+^, ${\mathrm{SO}}_{4}^{2-}$, and OH^−^ in the CPS on the Cl^−^ binding capacity. Additionally, the Cl^−^ binding isotherms were obtained for larger number of data points as compared to other binding isotherms in literature [8,31,32]. The binding isotherms from [25] for AFm and C-S-H in the hydrated cement paste (whose quantities are obtained from the microstructural modeling of concrete using VCCTL [22,23]) are utilized here to determine the bound Cl^−^ concentration based on the free Cl^−^ concentration (an output of the coupled transport model) and the pH of the CPS (given by the microstructural modeling of concrete in VCCTL [22,23]).

### 2.5. Composite Theory and Estimation of Material Diffusivity Parameters

**Figure 3:**
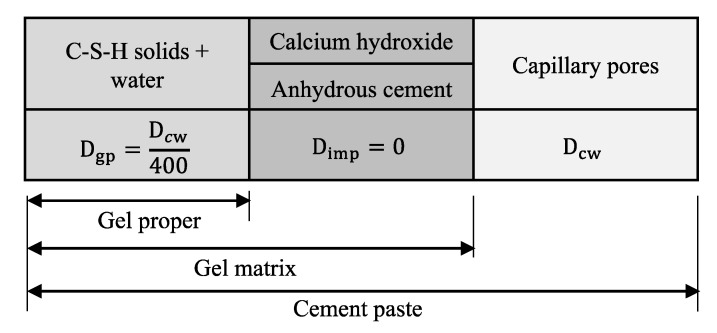
Diffusion coefficient for different components of cement paste.

The hydrated cement paste is assumed to be a composite of C-S-H, CH, gel water, capillary (pore) water, and anhydrous cement, as these constituents represent the major portion of the whole system. The composite theory for the cement paste system considers different components as separate entities for the transport of ionic species in concrete, as shown in Figure 3. The volume fraction of the components of the cement paste (C-S-H, CH, gel water, capillary (pore) water, and anhydrous cement) are obtained from microstructural modeling of concrete using VCCTL [22,23]. Anhydrous cement and CH are assumed to be impermeable entities for Cl^−^ diffusion (i.e., diffusivity equal to zero). The gel proper, which consists of gel water and gel solids, is assumed to have a bulk diffusivity, D_gp,_ which is taken to be 1/400 of the diffusivity of Cl^−^ in water, as experimentally observed by Garboczi and Bentz [33]. The diffusion coefficient in the capillary water, D_cw_, is taken to be the same as that of the bulk water at 298 K (=2.03 × 10^−9^ m^2^/s) [34]. Since temperature and RH in the concrete microstructure may not be the same as in the bulk water, correction terms are applied to the Cl^−^ diffusion in capillary water according to

(21) $$D_{\mathrm{cw}}=2.03\times 10^{-9}\times \exp\left(\frac{{\Delta U}_{{\mathrm{Cl}}_{Diff}}}{R}\left(\frac{1}{298}-\frac{1}{T}\right)\right)\times {\left(1+\frac{{\left(1-\mathrm{RH}\right)}^{4}}{{\left(1-{\mathrm{RH}}_{c}\right)}^{4}}\right)}^{-1}$$

where Δ$U_{{\mathrm{Cl}}_{Diff}}$ is the activation energy for diffusion of Cl^−^ in water, and R is the gas constant. Jensen [34] used the Maxwell equation to estimate the diffusion coefficient of the gel matrix, which is composed of CH, C-S-H, and anhydrous cement. To apply the Maxwell equation for a two-phase composite model, the CH and the anhydrous cement are considered discrete periodically spaced spheres (or inclusions) in the gel proper. The diffusion coefficient in the gel matrix, D_gm_, as shown in Figure 3, is then obtained from

D_gm_ = D_gp_ × V_gp_/(V_gp_ + 1.5 × (V_ce_ + V_CH_)) (22)

where V_gp_, V_ce_, and V_CH_ are the volume fractions of the gel proper, anhydrous cement and CH, respectively. The geometry of the capillary pore system of the cement paste is best described by the phase symmetric crumbled foil composites [16]. Hence, the diffusion coefficient for this composite system, i.e., the cement paste, D_p_, is given by

(23) $$D_{p}=D_{\mathrm{gm}}\times (n+2\times \sqrt{n}\times c\times (n-1))/(n+2\times \sqrt{n}-c\;(n-1))$$

where n = D_cw_/D_gm_, c is the capillary porosity of the composite (=V_cw_/(V_cw_ + V_gp_ + V_ce_ + V_CH_)), and V_cw_ is the volume fraction of the capillary water in the cement. Hence, the diffusion coefficient, D_i_, through concrete (a composite of hydrated cement paste, air, and aggregate) is given by

D_i_ = D_p_ × (1 − ϕ)/(1 + 0.5 × ϕ) (24)

where ϕ is the volume fraction of aggregate. Since aggregate-to-cement (a/c) ratio is commonly reported in experimental studies, the volume fraction of the aggregate, ϕ_agg_, is obtained from the a/c ratio according to

ϕ_agg_ = (a/c × ρ_cm_/ρ_agg_)/(1 + w/c × ρ_cm_/ρ_w_ + a/c × ρ_cm_/ρ_agg_) (25)

where ρ_cm_ is the density of cement (=3150 g/L), ρ_agg_ is the density of aggregate (=2650 g/L), and ρ_w_ is the density of water (=1000 g/L).

Schutter and Taerwe [35] found that the thermal diffusivity (or heat diffusion coefficient), D_T_, of concrete decreases linearly with the degree of hydration. Another study [36] established that the thermal diffusivity remains constant during the early hydration of the concrete; however, it decreases as much as 21% in mature concrete. In this study, the thermal diffusivity is assumed to decrease linearly with the degree of hydration of cement, r, (as obtained from microstructural development of concrete using VCCTL [22,23]) according to [35]

D_T_ = 0.004 × (1.1 − 0.1 × r) (26)

Similarly, the temperature-dependent RH diffusion coefficient is obtained using the formulation in Bazant and Najjar [37] as follows

D_RH_(RH, T) = D_Tref_ × f_T_ (27)

(28) $$D_{\mathrm{Tref}}=(0.25\;[\frac{cm^{2}}{\mathrm{day}}]\;\left(f_{1}+\frac{\left(1-f_{1}\right)}{1+{f_{2}}^{f_{3}}}\right))$$

(29) $$f_{T}=\frac{T}{T_{\mathrm{ref}}}\times \exp\left(\frac{{\Delta U}_{{\mathrm{RH}}_{Diff}}}{R}\left(\frac{1}{T_{\mathrm{ref}}}-\frac{1}{T}\right)\right)$$

where f_T_ is a parameter to account for the temperature on D_RH_, Δ$U_{{\mathrm{RH}}_{Diff}}=38.91\;\mathrm{kJ}/\mathrm{mol}$ is the activation energy for diffusion, D_Tref_ is the RH diffusion coefficient at the reference temperature, f_1_ is taken equal to 0.05 for concrete [3], $f_{2}=\frac{\left(1-\mathrm{RH}\right)}{\left(1-{\mathrm{RH}}_{c}\right)}$, $f_{3}$ is an exponent to characterize the extent of drop in diffusivity at critical RH in concrete (taken equal to 16 according to [3]), and ${\mathrm{RH}}_{c}$ is a critical RH at which the diffusion coefficient for RH faces a sudden drop [38]. RH_c_ is taken as 0.75 as observed by Bazant and Najjar [38]. Isgor and Rzaqpur [37] demonstrated that the transport of O_2_ depends on the degree of pore saturation as O_2_ can also penetrate in concrete through both air and pore solution. Papadakis et al. [39] proposed the following for the estimation of the O_2_ diffusion coefficient

(30) $$D_{O_{2}}=1.92\times 10^{-6}\;\mu_{p}(t)\times (1-\mathrm{RH}{)}^{2.2}$$

where μ_p_(t) is the porosity of concrete, which is obtained from microstructural development of concrete using VCCTL [22,23].

### 2.6. Implementation, Verification, and Sensitivity Studies

As mentioned earlier, the model developed here is implemented in MOOSE [26] according to the flow chart shown in Figure 1. In MOOSE, four kernels (heat, RH, Cl^−^, and O_2_ diffusion) were created to solve the weak form of the governing equations for heat, RH, O_2_, and Cl^−^ diffusion as indicated in Equations (4)–(6) and (10), respectively. MOOSE solves these four diffusion equations in the respective kernels. Additionally, two classes (material and boundary condition) were created. The diffusion coefficients for heat, RH, Cl^−^, and O_2_ are estimated in the material class, and boundary condition for each variable in the governing equations are applied in the boundary conditions class. The concrete domain geometry, the mesh information, and the external environmental conditions (temperature and RH) on the concrete boundary during curing and exposure periods are input in MOOSE through an input file. Moreover, the microstructural properties from VCCTL [22,23] for the entire duration of the analysis are input in MOOSE at the beginning of the analysis. The microstructural properties (volume fraction of hydrated components (AFm, C-S-H, and CH), anhydrous cement, volume fraction of pore water, porosity, degree of hydration of cement, and pH of the CPS) are obtained in the material class in a given time step and for each node based on the input VCCTL data at the corresponding equivalent maturation time using the temperature and RH values at that location. The material and boundary conditions class and the input file are both ways connected with the heat, RH, Cl^−^, and O_2_ diffusion kernels to access the required parameters. Apart from the accessibility of the material parameters, the RH diffusion kernel uses temperature as a coupled variable and the Cl^−^ diffusion kernel uses RH as a coupled variable. The model outputs the time-dependent temperature, RH, Cl^−^, and O_2_ profiles in the concrete domain. MOOSE solves the transient coupled transport problem within a finite element framework by employing a preconditioned Jacobian-Free Newton–Krylov (PJFNK) method.

**Figure 4:**
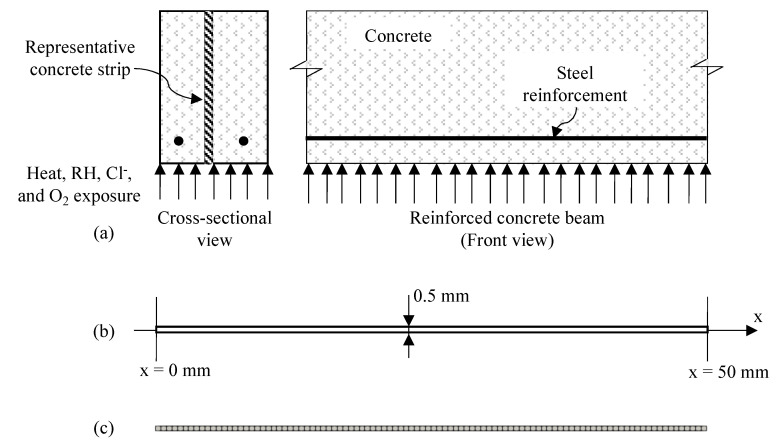
(**a**) Representative concrete strip for 1-D transport in RC beam problem; (**b**) concrete strip model; (**c**) meshing of the concrete strip.

**Figure 5:**
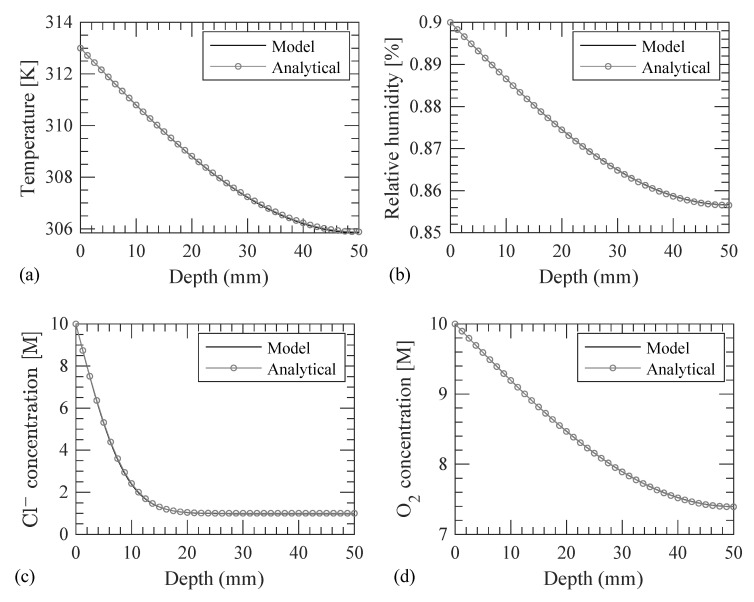
Comparison of results from model and analytical solution of (**a**) temperature in case 1, (**b**) RH in case 2, (**c**) Cl^−^ in case 3, and (**d**) O_2_ in case 4.

The implementation is verified using a concrete strip, which is representative of the cross-section of a RC beam exposed to heat, RH, Cl^−^, and O_2_, as show in Figure 4a. The concrete strip is, as shown in Figure 4b, discretized into 100 finite elements as shown in Figure 4c. The modeling parameters are presented in Table 1. The modeling is verified by simplifying the parameters of the governing equations and the boundary and initial conditions into four cases, as indicated in Table 2. Analytical solution (as presented in [40]) for temperature, RH, Cl^−^, and O_2_ are obtained for each case. Results from the modeling of all four cases matched with the analytical solution as shown in Figure 5. Therefore, the model is verified for diffusion of heat, RH, Cl^−^, and O_2_ with known parameters.

**Figure 6:**
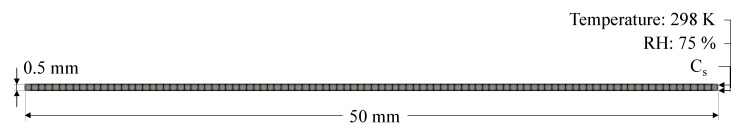
Two-dimensional concrete strip for sensitivity analysis.

A sensitivity analysis is also performed using the same 1D diffusion problem described above for the implementation verification and illustrated in Figure 4. The objective of the sensitivity analysis is to identify the key variables that affect the Cl^−^ profile in the concrete domain, particularly within the realizations of the model developed here. Cl^−^ are considered to transport from the left end of the strip as indicated in Figure 6. Boundary conditions for temperature and RH are also shown in Figure 6. The Cl^−^ concentration profile is obtained in concrete by varying one parameter at a time from its base value as indicated in Table 3. The concrete properties (cement type, aggregate type, w/c ratio, a/c ratio, and air content), parameters of the curing and exposure conditions (temperature and RH), boundary conditions (exposure zone, and temporal variation function for surface Cl^−^ concentration), and parameters of the diffusion equations (Cl^−^ diffusivity in water, critical RH, and activation energy for cement hydration, Cl^−^ diffusion in capillary water, and RH diffusion) are considered in the sensitivity analysis. Variations in the cement type and aggregate type are considered to study the effect of moisture capacity of cement paste and aggregate on the diffused Cl^−^ concentration in the concrete domain. The variation in concrete mix proportions (w/c ratio, air content, a/c ratio), temperature and RH conditions during curing and exposure are taken based on experience and existing literature [41]. Variations in critical RH are taken based on [42]. Surface Cl^−^ concentration is varied based on the experimental data in tidal, atmospheric, and spray zones, as reported in [4]. Other modeling parameters (Cl^−^ diffusion coefficient in capillary water at 298 K, activation energy for hydration of cement and diffusion of Cl^−^ and RH) are varied by ±25% of their base values due to lack of information. It should also be noted here that, in reality, each one of these variables can be estimated with a different degree of certainty. For example, it is inherently more difficult to estimate the time varying surface Cl^−^ concentration, curing duration, and temperature and RH during curing. This sensitivity analysis uses a somewhat arbitrary difference from the base values based on engineering judgment and data in literature and does not necessarily extend into this varying uncertainty from parameter to parameter.

**Figure 7:**
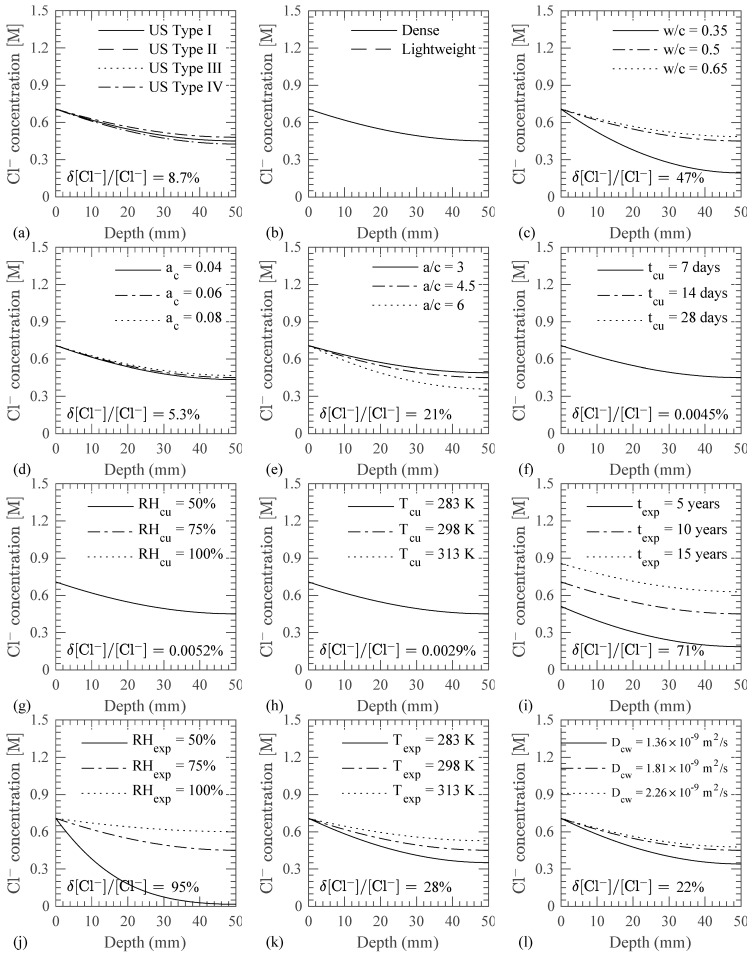

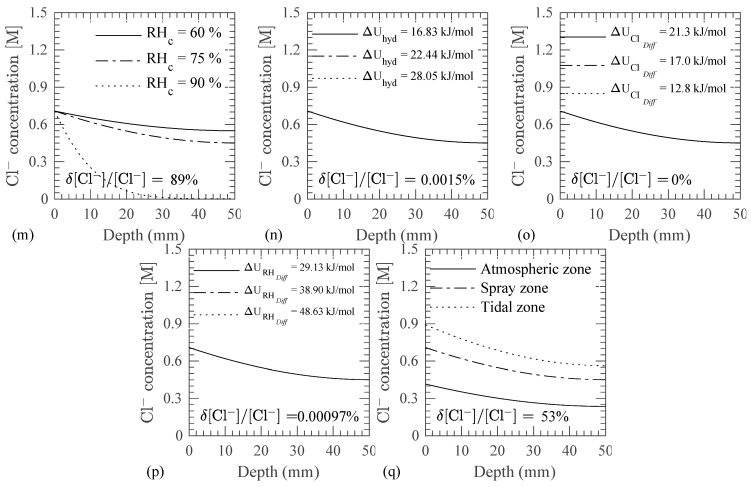
Effect on Cl^−^ transport of varying (**a**) cement type; (**b**) aggregate type; (**c**) w/c ratio; (**d**) air content, a_c_; (**e**) aggregate-to-cement (a/c) ratio; (**f**) curing duration, t_cu_; (**g**) RH during curing, RH_cu_; (**h**) temperature during curing, T_cu_; (**i**) exposure duration, t_exp_; (**j**) RH during exposure, RH_exp_; (**k**) temperature during exposure, T_exp_; (**l**) Cl^−^ diffusivity in capillary water at 298 K, D_cw_; (**m**) critical RH, RH_c_; (**n**) activation energy for cement hydration, ΔU_hyd_; (**o**) activation energy for Cl^−^ diffusion, Δ$U_{{\mathrm{Cl}}_{Diff}}$; (**p**) activation energy for RH diffusion, Δ$U_{{\mathrm{RH}}_{Diff}}$; and (**q**) exposure zone with different surface Cl^−^ concentration.

The effect of variation in the parameters on the diffused Cl^−^ profile in the concrete domain, as shown in Figure 7, is quantified by determining the difference in the diffused Cl^−^ concentration and normalizing by that corresponding to the base case, i.e., δ[Cl^−^]/[Cl^−^], at a depth of 50 mm from the concrete surface according to

δ[Cl^−^]/[Cl^−^] = |[Cl^−^]_max_ − [Cl^−^]_min_|/[Cl^−^]_b_ (31)

where [Cl^−^]_max_ and [Cl^−^]_min_ are the maximum and minimum Cl^−^ concentrations for the considered values of the parameter and [Cl^−^]_b_ is the Cl^−^ concentration for the base case of that parameter. The following observations are made from the sensitivity study:

- As shown in Figure 7a,b, the cement and aggregate type affect the Cl^−^ profile in the concrete through the water adsorption isotherms for cement paste and aggregate, as described in Section 2.4. The water adsorption isotherms for given aggregate and cement type affect the moisture capacity of cement paste and aggregate. However, the use of dense or lightweight aggregates did not have a significant impact on the Cl^−^ transport profile for the test case.
- As shown in Figure 7c, the w/c ratio strongly influences the Cl^−^ transport which is due to a change in the concrete porosity with changing w/c ratio. A highly porous concrete microstructure due to a higher w/c ratio increases the capillary pores and results in a faster diffusion of Cl^−^ in the concrete as compared to the cases with a low w/c ratio.
- As seen in Figure 7d, the Cl^−^ concentration in the concrete increases as the air content increases in the concrete. A higher air content increases the capillary porosity and hence the Cl^−^ diffusion coefficient of the cement paste as per Equation (23).
- The effect of the aggregate content on Cl^−^ diffusion is illustrated in Figure 7e. Since the aggregates are considered impermeable for Cl^−^ transport, the Cl^−^ diffusion coefficient reduces as the aggregate content increases.
- Curing duration and environment (temperature and RH) affect the development of the concrete microstructure, which results in different Cl^−^ profiles in concrete. However, in this study, while taking into consideration the variation in the curing duration, the environmental conditions during curing and exposure periods are assumed to be the same, which resulted in insignificant differences in the Cl^−^ concentration profiles in concrete as shown in Figure 7f.
- Similarly, the individual variation of temperature and RH during curing is found to have a little impact on the Cl^−^ concentration profiles as seen in Figure 7g,h. This is due to the larger exposure period, i.e., 10 years, as compared to short curing period, i.e., 14 days.
- As shown in Figure 7i, the exposure duration affects the Cl^−^ concentration in the concrete because more salt solution diffuses inside concrete as the exposure duration increases with constant environmental conditions and material parameters.
- As seen in Figure 7j, the variation in the RH during exposure period affects the Cl^−^ transport in concrete. For 50% RH, which is less than the critical RH, i.e., 75%, negligible Cl^−^ transport is observed as compared to 75% and 100% RH cases. This is due to a reduction in the RH diffusion coefficient as per Equations (27)–(29). Reduction in the RH diffusion coefficient results in a reduced rate of transport of RH. Since Cl^−^ transport is coupled with RH transport (refer to Equations (10) and (21)), a reduced Cl^−^ concentration is observed.
- As shown in Figure 7k, the Cl^−^ transport is also affected from temperature during the exposure period. Since the variation in the temperature affects the RH transport (refer to Equations (27)–(29)), the degree of hydration (refer to Equation (13)), and the Cl^−^ diffusion coefficient in the capillary water (refer to Equation (21)), a higher Cl^−^ concentration is observed at the higher exposure temperature.
- As demonstrated in Figure 7l, the diffusion coefficient of Cl^−^ in the capillary water significantly affects the Cl^−^ transport in the concrete as per Equation (21). The higher diffusion coefficient in the capillary water results in a faster diffusion of Cl^−^ in the concrete microstructure.
- As observed earlier in Figure 7j, a RH below the critical RH results in slower diffusion of Cl^−^ in concrete, a similar trend is observed for the variation of the critical RH in the concrete microstructure as shown in Figure 7m. A higher critical RH causes more RH exposure below the critical RH, which results in a slower diffusion of RH as compared to cases with a lower critical RH. Thus, a lower Cl^−^ concentration profile is observed for the cases with a higher critical RH as compared to the cases with a lower critical RH.
- The activation energy for hydration, Cl^−^ diffusion, and RH diffusion are found to have a negligible impact on the Cl^−^ concentration profile as shown in Figure 7n–p, respectively. Equal curing and exposure conditions result in the low sensitivity of the model to these parameters (as compared to other parameters) as per Equation (13), Equation (21), and Equation (29).
- The evolution of surface Cl^−^ affects the Cl^−^ concentration profile in the concrete as seen in Figure 7q. The evolution of the surface Cl^−^ in an atmospheric zone is slower as compared to tidal and spray zones as indicated in Table 3. Therefore, a higher Cl^−^ concentration in the concrete is observed in tidal and spray zones as compared to an atmospheric zone.

### 2.7. Validation

**Figure 8:**
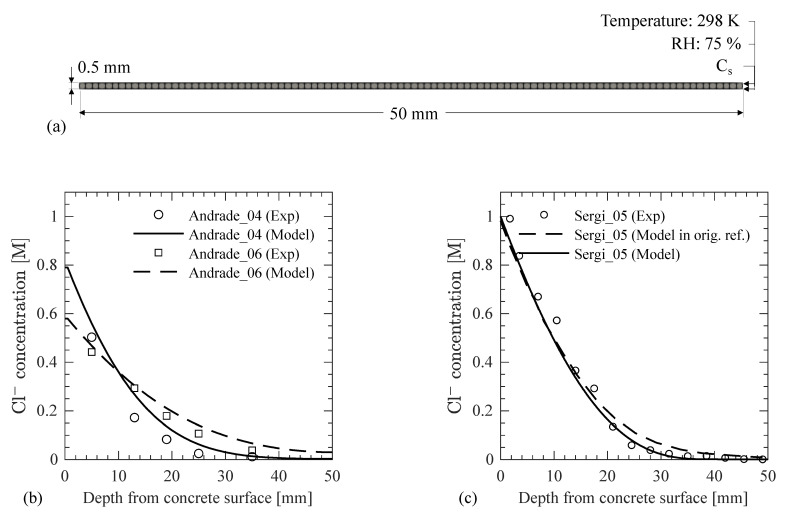
(**a**) Numerical model for validation study, and comparison of the simulation results with experimental data from (**b**) Andrade et al. [11] and (**c**) Sergi et al. [43].

In this section, the predictive ability of the developed model for Cl^−^ transport in concrete is examined with data from different experimental studies in literature. Experimental studies that reported most of the required information for the developed modeling framework were selected for the validation purpose. Andrade et al. [11] studied the transport of Cl^−^ in concretes with and without admixtures. Here the comparison is limited to concretes with no admixtures because the concrete microstructure development approach is not validated for the effects of water-reducing or other admixtures. Similarly, Sergi et al. [43] conducted experiments to study the Cl^−^ diffusion in concrete and also developed a model for the same purpose. The mixture proportions of the concrete specimens and other details from [11,43] are given in Table 4. Andrade et al. [11] used the AASTHO T 259 [44], 90-day ponding test, for the Cl^−^ transport study, whereas Sergi et al. [43] exposed cement paste cylinders to 1 M NaCl solution for 100 days. Two salt ponding experimental studies for w/c = 0.4 and 0.6 are denoted as Andrade_04 and Andrade_06, whereas the salt exposure tests on the cement paste by [43] is denoted as Sergi_05 in Table 4. The parameters that were used in the modeling are also provided in Table 4. The surface Cl^−^ concentration is assumed to increase logarithmically with time from zero to its ultimate value at the end of the tests, which is provided in the original references. Andrade et al. [44] performed ponding tests on 600 (length) × 300 (width) × 150 (depth) mm slab specimens while Sergi et al. [43] used cylinder specimens with a 49 mm diameter and a 75 mm length. Andrade et al. [44] extracted 100 mm diameter cores from the slab for examining the Cl^−^ concentration. Sergi et al. [43] sliced off 10 cm from the cast ends and masked other surfaces with paraffin wax to allow Cl^−^ diffusion from one end only. In both studies, the Cl^−^ concentration was not measured beyond 50 mm from the concrete surface due to a limited diffusion of Cl^−^. Thus, by taking advantage of symmetry, specimen geometry was simulated using a concrete strip as shown in Figure 8a.

Results after 90 days of salt ponding test for Andrade_04 and Andrade_06 are compared with the numerical model in Figure 8b. The results matched the experimental data with a coefficient of determination, R^2^, value of 0.90 for w/c = 0.4 and 0.97 for w/c = 0.6. It is noted here that the Cl^−^ diffusion coefficient in the capillary water is assumed based on previous research [34] as given in Table 4. Similarly, the a/c ratio is assumed based on experience, and the results of the sensitivity analysis above show that a change in a/c ratio may affect the Cl^−^ concentration. Simulation results for Sergi_05 are compared with the experimental results and the model developed by Sergi et al. [43]. The developed model estimates the Cl^−^ concentration with an R^2^ value of 0.98. Even though the surface Cl^−^ concentration, w/c ratio, and air content were different in the two experiments, it is seen that an accurate characterization of the microstructural properties of concrete and a consideration of the environmental conditions during curing and exposure in the modeling provide a reliable estimate of the Cl^−^ profile in the concrete domain. It is concluded here that the developed model can accurately estimate the Cl^−^ profiles in concretes with different mixture proportions and curing and exposure conditions. It is also noted that the results can be further improved if more data on model parameters is collected from the experiments, particularly, on the temporal variation of surface Cl^−^ concentration, which is an essential boundary condition of the numerical model and largely influences the simulation results as demonstrated in the sensitivity analysis.

## 3. Case Study and Discussion

**Figure 9:**
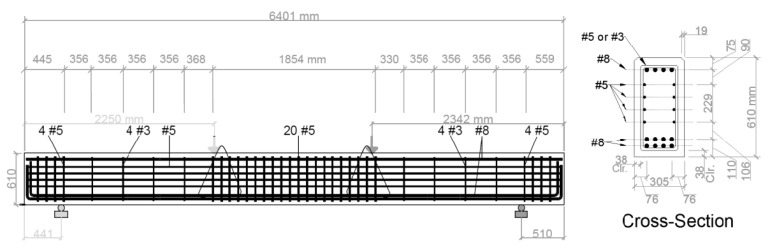
Dimensions and reinforcement details of the RC beam used as a case study (the rebars diameters are indicated as per the U.S. system, e.g., #3, #5, etc.)

**Figure 10:**
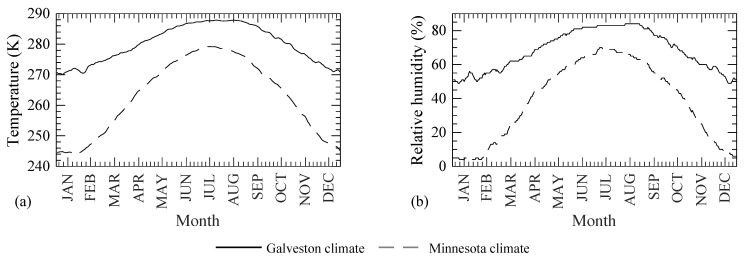
Average weekly temporal variation of (**a**) temperature and (**b**) RH in Galveston, Texas, and North Minnesota, Minnesota (data from [45]).

**Figure 11:**
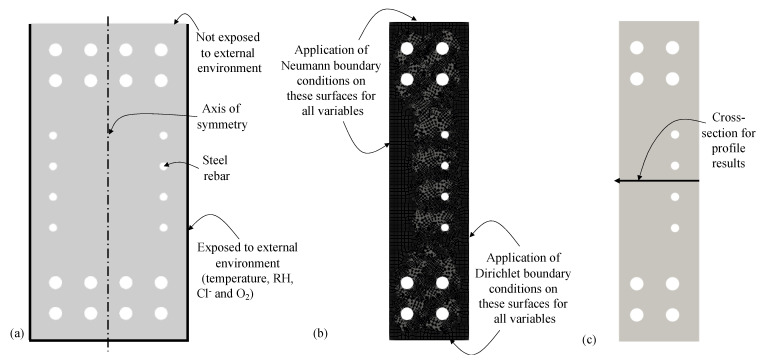
(**a**) Model of the cross-section of the beam, (**b**) meshed geometry, and (**c**) cross-section along which the comparison of results is performed in the concrete domain.

To demonstrate the full capabilities of the developed model beyond the 1D diffusion studies performed in controlled laboratory environments, the Cl^−^ transport in an RC beam located in two different climates, Galveston, Texas (warm and humid), and North Minnesota, Minnesota (cold and dry), is simulated as a case study in this section. The elevation view and the cross-sectional properties of the beam are shown in Figure 9. The concrete mixture proportions are taken similar to those in the base case of the sensitivity study presented in Section 2.6. The concrete mixture details along with the modeling parameters are given in Table 5 for completeness. The beam is assumed to be cured in and exposed to the environmental conditions in the aforementioned two regions (Galveston and North Minnesota). The temperature and RH profiles for both regions are shown in Figure 10, which were obtained from Iowa Environmental Mesonet [45]. The temporal climate data are used repeatedly after a year of exposure. Additionally, the same airborne Cl^−^, for both regions, is assumed to accumulate on the three surfaces (indicated with solid black lines) of the beam in Figure 11a. The Cl^−^ source is assumed to be from the Gulf in the coastal Galveston and from deicing salts in North Minnesota. To enable a comparison, an identical evolution of the surface Cl^−^ concentration, based on the experimental data in [4] measured on a dockyard in a marine environment, is assumed as

C_s_ [g/g of concrete] = 0.0021 × (t/365)^0.47^ (32)

where *t* is time in days. Since the Cl^−^ binding isotherm utilizes the free Cl^−^ concentration in mol/L of pore solution, the Cl^−^ concentration in g/g of concrete is converted into mol/L of pore solution at each node in the concrete domain according to

C_f_ [mol/L] = C_s_ [g/g of concrete] × ρ_conc_ [g/L]/(35.45 [g/mol] × f_cw_ [L/L]) (33)

where f_cw_ is the volume fraction of the capillary water in the concrete, and ρ_conc_ is the density of concrete evaluated according to

ρ_conc_ = (1 + w/c + a/c) × ρ_cm_/(1 + w/c × ρ_cm_/ρ_w_ + a/c × ρ_cm_/ρ_agg_) (34)

Since the longitudinal reinforcement is uniform along the length of the beam, a 2D model is used to study the coupled transport process as shown in Figure 11a while the modeling approach and the implementation are capable of analyzing 3D problems. Since the model and boundary conditions are symmetric about the vertical axis (as indicated in Figure 11a), only one half of the cross-section is used in the finite element analysis in MOOSE as shown in Figure 11b. The steel reinforcing bars are considered impermeable for RH, Cl^−^, and O_2_ diffusion. The results from the simulations are compared, later in this section, along the line shown in Figure 11c. The O_2_ concentration on the concrete surface and the O_2_ initially dissolved in the CPS are assumed to be 0.0085 kg/m^3^ and 0.005 kg/m^3^, respectively, based on [46].

**Figure 12:**
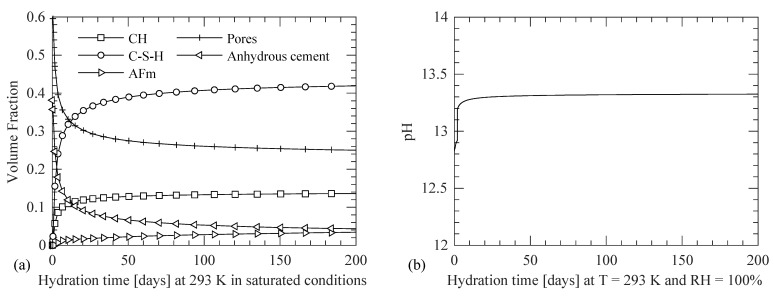
Evolution of (**a**) cement paste hydration products (CH: calcium hydroxide, C-S-H: calcium-silicate-hydrate, and AFm: mono-sulfate aluminate) and (**b**) pH of CPS at 293 K and 100% RH.

The concrete is hydrated isothermally at 293 K in saturated conditions to obtain the temporal evolution of the components of the cement paste in concrete using VCCTL [22,23] as shown in Figure 12a. The evolution of pH is also shown in Figure 12b. The evolution of the concrete microstructure and pH is shown until 200 days after which the changes in the values become insignificant. The microstructural properties are input in the finite element model of the beam in MOOSE and used accordingly based on the equivalent maturation time concept described earlier. Due to the higher heat transfer coefficient of steel and one week as a time step for the analysis, the temperature profile in the concrete domain is assumed to be independent of the heat transfer through the steel reinforcement. One week is selected as a sufficiently small-time step to account for the effect of temporal environmental conditions (temperature and RH) on the transport of RH, Cl^−^, and O_2_. As described earlier in Figure 1, the microstructural properties from VCCTL [22,23] are utilized at each node in the concrete domain for the corresponding equivalent maturation time based on the temperature and RH of those nodes. To demonstrate the importance of considering the temporal variations in the microstructural properties of concrete due to changing environmental conditions, results are compared against a case in which the equivalent maturation time is set to 28 days for the whole duration of analysis and for the entire concrete domain. Thus, the microstructural properties do not update as the concrete maturates with time; however, the transport properties still include the effect of spatial variation of temperature and RH, as described in Section 2.4 and Section 2.5 and Equations (15)–(30). In both climatic regions, the simulation using the equivalent maturation time as an independent variable is labeled as Case 1 and that with a set 28 days equivalent maturation time is labeled as Case 2.

**Figure 13:**
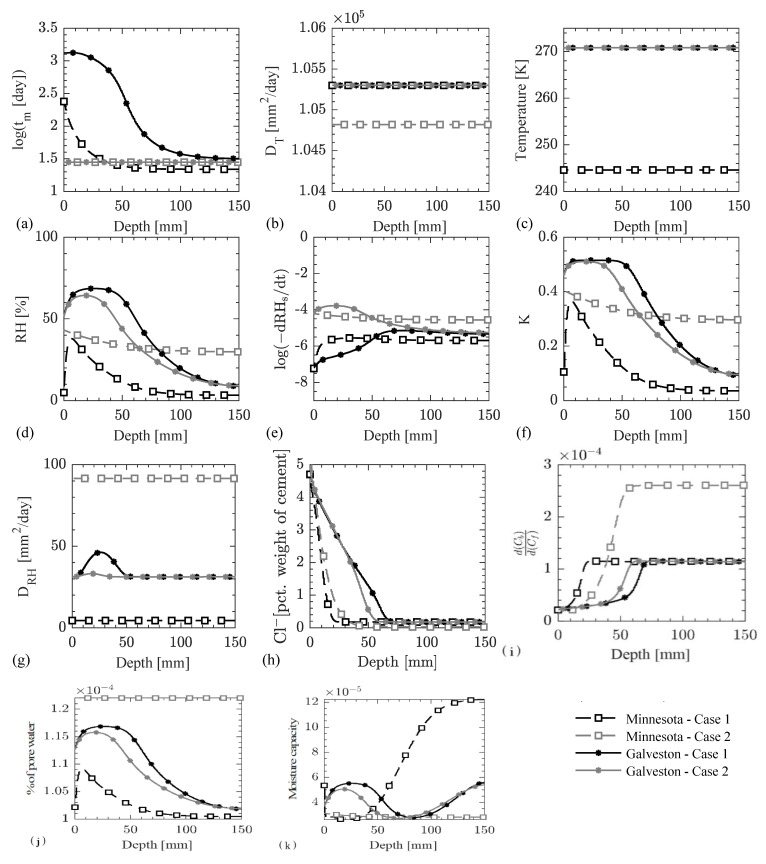
Profiles at the end of 10 years of exposure across concrete depth of (**a**) equivalent maturation time; (**b**) heat diffusion coefficient; (**c**) temperature; (**d**) RH profile; (**e**) rate of self-desiccation of RH, $\frac{dRH_{s}}{dt}$; (**f**) coupling term with heat diffusion in governing equation for RH diffusion, K; (**g**) RH diffusion coefficient; (**h**) Cl^−^ concentration; (**i**) Cl^−^ diffusion coefficient; (**j**) bound Cl^−^ concentration; and (**k**) % pore water.

As shown in Figure 13, the results for Case 1 and Case 2 at the end of 10 years of exposure in two different climatic regions are compared along a line in the cross-section of the beam, which is shown in Figure 11c. The following observations are made from this case study.

- It is clearly seen in Figure 13a that the concrete maturation time is enforced as 28 days for Case 2 in both climatic regions for the entire duration of the analysis. The equivalent maturation time for Case 1 is found to be larger in Galveston as compared to North Minnesota due to warm and humid environmental conditions in the former (refer to Figure 10). Since the equivalent maturation time is kept constant for Case 2, only the effects of temporal variation of temperature and RH on the modeling parameters are reflected. Case 1, on the other hand, accounts for both the time varying temperature and RH and the temporal changes in the microstructural properties.
- Figure 13b shows the variation of the thermal diffusivity along depth of the beam. Since the temperature diffusion coefficient depends on the degree of hydration as per Equation (26) (which is a function of the equivalent maturation time) and the equivalent maturation time is more than 21 days (as shown in Figure 13a), no significant change in the temperature diffusion coefficient is observed. The high thermal diffusivity and a time step of seven days used in the simulations resulted in an almost uniform temperature distribution in the concrete domain (see Figure 13c), which is approximately equal to the temperature on the concrete surface at the end of the year as shown in Figure 10.
- The variation in the RH profile in concrete is shown in Figure 13d. The RH value at the concrete surface depends on the RH value in two climatic zones in January (or at the beginning of a simulation year as shown in Figure 10). The RH value in the concrete domain depends on the history of RH values, rate of self-desiccation of RH, coupling term of RH diffusion with temperature, and the RH diffusion coefficient. Since the RH values at the concrete surface peaks in between June and August of each year, that peak travels in the concrete domain and appears at a certain depth in January. The peak diffuses more in Galveston as compared to North Minnesota due to colder and drier climate in the latter, which affects the rate of self-desiccation of RH (based on Equation (5)), coupling term with temperature diffusion (based on Equation (5)), and the diffusion coefficient for RH (based on Equation (27)).
- The instantaneous profile of rate of self-desiccation of RH in concrete domain at the end of 10 years, see Figure 13e, is governed by the temperature, RH, and the equivalent maturation time profiles in the concrete domain based on Equation (5). The rate of self-desiccation of RH is observed to be higher in Case 1 as compared to Case 2 in both climatic zones due to a higher equivalent maturation time. The instantaneous profiles for the coupling term and RH diffusion coefficient in the concrete domain at the end of 10 years (see respectively Figure 13f,g) depend on the RH profile (based on Equations (5) and (27)). Both achieve their peak values around the same depth with the peak of RH profile for both cases and climatic regions.
- As seen in Figure 13h, more Cl^−^ diffused in Galveston as compared to North Minnesota due to a colder and drier climatic condition in the latter, which causes a lower temperature and RH profile in the concrete domain and a lower maturity and porosity in the concrete. Additionally, the drier climate in North Minnesota causes RH to be below the critical RH of 75%, which drastically reduces the Cl^−^ diffusion as shown in the sensitivity study; see Figure 7m.
- The Cl^−^ diffusion coefficient profile shown in Figure 13i follows the trend of the pore water content; see Figure 13k. As the concrete maturity decreases along the depth, as seen in Figure 13a, the weight fractions of C-S-H and AFm, and the pH of the CPS also decrease (see Figure 12a,b), which results in an increase in the binding capacity of the Cl^−^ as shown in Figure 13j. Additionally, the decrease in the free Cl^−^ concentration (see Figure 13h) contributes to the increase in the bound Cl^−^ concentration, i.e., more Cl^−^ per unit mole of free Cl^−^ bound to the concrete surface at lower free Cl^−^ concentrations as compared to higher free Cl^−^ concentrations due to the presence of larger surface area of C-S-H and more binding sites on the AFm phases. The lower bound Cl^−^ concentration in North Minnesota as compared to Galveston is due to the lower concrete maturity and the lower free Cl^−^ concentration as per Figure 13a,h, respectively. The highest binding capacity is observed for Case 2 in North Minnesota climate, which is due to a combination of lower pH values (as a result of lower equivalent maturation time) and a lower free Cl^−^ concentration as compared to the other three simulation cases.
- As discussed earlier, the concrete maturity decreases along the concrete depth which increases the concrete porosity based on Figure 12a. However, the RH decreases along the concrete depth as observed in Figure 13d, which determines the amount of water in these pores, and therefore, the pore water decreases along the concrete depth as seen in Figure 13k.

**Figure 14:**
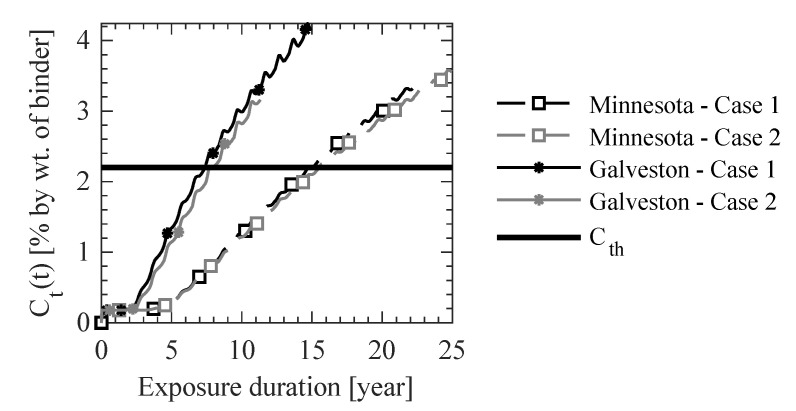
Temporal Cl^−^ profiles in the concrete domain at the steel-concrete interface.

Sandberg et al. [47] reported that the threshold Cl^−^ concentration for outdoor structures varies from 0.17% to 2.2% by weight of cement. The evolution of the Cl^−^ concentration at the steel-concrete interface is plotted against time in Figure 14 to evaluate the time for corrosion initiation. The Cl^−^ concentration is found to exceed a threshold Cl^−^ concentration of 2.2% by weight of cement at the steel concrete interface in 14.77 years for Case 1 and 15.58 years for Case 2 in North Minnesota and in 7.38 years for Case 1 and 7.79 years for Case 2 in Galveston as shown in Figure 14. The difference in the estimation of the corrosion initiation for the two climatic regions between the two cases is about 5.4%. Additionally, the consideration of temporal microstructural properties of concrete results in differences in the predictions of RH, rate of self-desiccation of RH, coupling term of RH diffusion with heat diffusion, diffusion coefficient for RH, Cl^−^ concentration, and Cl^−^ diffusion coefficient for both climatic conditions. These differences are both time and location dependent, and they are observed to be as large as 480% for Cl^−^ concentration and 23.6% for RH after 10 years of exposure. Thus, the consideration of temporal microstructural properties along with the coupling of temperature and RH provides an improvement over the existing models in obtaining a more accurate description of the coupled transport of Cl^−^ in concrete.

## 4. Conclusions

In this study, modeling of coupled transport of heat, RH, Cl^−^, and O_2_ is performed by incorporating concrete microstructural properties of hardened concrete, temperature, and RH conditions during curing and exposure periods, empirically derived Cl^−^ binding isotherms, analytical water adsorption isotherms and the composite theory. The constraint of simulating concrete microstructure in specific temperature and RH conditions, i.e., isothermal and adiabatic conditions for sealed and completely saturated cases, is overcome by utilizing the concept of equivalent maturation time. Thus, the shortcomings of the existing numerical models [2,15,16,17,18,19] were addressed in this study for a more accurate prediction of the temperature, RH, Cl^−^, and O_2_ concentration in a concrete structure. The developed modeling framework is verified using analytical solutions of simplified diffusion problems. A sensitivity study is performed to identify the modeling parameters that affect the Cl^−^ concentration in the concrete domain. The modeling approach is validated for 1-D diffusion of Cl^−^ using existing experimental results and applied to a complex RC beam situated in the coastal environmental conditions of Galveston, Texas, and North Minnesota, Minnesota, to demonstrate the full model capabilities. Despite presence of a number of numerical models for Cl^−^ transport in concrete, the novelty of the developed numerical modeling framework and key findings from this study are

- The modeling framework is capable of accounting for the concrete material heterogeneity due to spatial and temporal distribution of temperature and RH conditions in the concrete domain to study the coupled transport problem. The temporal heterogenous concrete microstructure properties (volume fractions of C-S-H, CH, AFm, anhydrous cement, and pore water, and the pH of the CPS), temperature and RH profiles, water adsorption isotherms (dependent on temperature, RH, w/c ratio, type of cement and aggregates, fraction of cement and aggregates and pore water), Cl^−^ binding isotherms (dependent on Cl^−^ concentration, pH of the CPS, weight fractions of C-S-H and AFm), and composite theory (dependent on temperature, RH, a/c ratio, air content, and the fraction of pore water, C-S-H, CH, and anhydrous cement) are necessary to estimate the spatially and time varying material diffusivity parameters for heat, RH, Cl^−^, and O_2_ transport.
- From the sensitivity study, the w/c ratio, the exposure duration, the boundary conditions (temperature, RH, surface Cl^−^ concentration, Cl^−^ diffusion coefficient in the capillary water at 298 K, and the critical RH) are identified as key variables that affect the Cl^−^ concentration in the concrete domain. The sensitivity of these parameters ranges from 22% (for Cl^−^ diffusion coefficient in the capillary water at 298 K) to 95% (for RH during exposure period) for the analyzed case. It is important to obtain these parameters carefully to predict accurate Cl^−^ concentration fields in the concrete domain.
- The modeling framework was able to produce temporal and spatially varied concrete microstructure on a two-dimensional RC beam for given temperature and RH conditions. The obtained concrete microstructure was utilized to obtain the temperature, RH, and Cl^−^ and in the concrete domain. The consideration of temporal concrete microstructure properties could result in a difference of as high as 480% after 10 years of exposure in Cl^−^ concentration depending on the time and location of measurement along the depth of the concrete domain.
- The model could predict the temporal and spatial variation in the diffusion coefficients for RH, Cl^−^, and O_2_ in the concrete domain. Thus, the model can be utilized further to simulate the corrosion reactions of steel reinforcement that uses temporal variations in Cl^−^ and O_2_ concentrations, temperature RH, and pH of the CPS. Furthermore, the degradation of RC structures due to Cl^−^ induced corrosion of steel reinforcement can be studied by accounting for the temporal and spatial variations in the environmental conditions and microstructural properties of concrete.
- Further research is required to experimentally validate the microstructure development for concretes with SCMs. The proposed model can be further be improved to include the effect of SCMs on the transport of heat, RH, Cl^−^, and O_2_ in the concrete domain for concretes with SCMs, which are becoming more prevalent in the construction industry.

## Figures and Tables

**Table 1:** List of parameters and their values for verification study.

Parameters	Value
Cement type	ASTM Type I
Coarse aggregate type	Dense crushed limestone
Water-to-cement (w/c) ratio	0.4
Air content, a_c_ [%]	7.9
Tri-calcium silicate (C_3_S) [%]	59.8
Di-calcium silicate (C_2_S) [%]	20.7
Tri-calcium aluminate (C_3_A) [%]	8.1
Tetra-calcium aluminoferrite (C_4_AF) [%]	11.5
Aggregate-to-cement (a/c) ratio	1.9
Curing duration, t_cu_ [days]	14
RH during curing, RH_cu_ [%]	100
Temperature during curing, T_cu_ [K]	296
Sample age at the start of experiment [days]	104
Exposure duration, t_exp_ [days]	90
RH during exposure, RH_exp_ [%]	100
Temperature during exposure, T_exp_ [K]	296
Cl^−^ diffusion coefficient in capillary water at 298 K, D_cw_ [m^2^/s]	2.03 × 10^−9^

**Table 2:** Initial and boundary conditions and modeling parameters for verification study.

Modeling Assumptions		Case 1	Case 2	Case 3	Case 4
Initial conditions	T (x, t = 0) [K]	298	298	298	298
	RH (x, t = 0) [%]	90	75	90	90
	Cl^−^ (x, t = 0) [M]	1.0	1.0	1.0	1.0
	O_2_ (x, t = 0) [M]	1.0	1.0	1.0	1.0
Boundary conditions	T (x = 0, t) [K]	313	298	298	298
	RH (x = 0, t) [%]	90	90	90	90
	Cl^−^ (x = 0, t) [M]	1.0	1.0	10.0	1.0
	O_2_ (x = 0, t) [M]	1.0	1.0	1.0	10.0
Parameters	D_T_ [mm^2^/day]	1 × 10^5^	*	*	*
	D_RH_ [mm^2^/day]	*	30	*	*
	D_i_ [mm^2^/day]	*	*	0.5	*
	D_O2_ [mm^2^/day]	*	*	*	300
	d(RHs)/dt [1/day]	*	0	*	*

* Values are obtained based on the modeling parameters listed in Table 1, and they vary during the simulation.

**Table 3:** List of parameters and their values for sensitivity analysis.

Parameters	Base Value	Values for Sensitivity Analysis
Cement type	US Type I	US Type II, III, and IV
Aggregate type	Dense	Lightweight
Water-cement (w/c) ratio	0.50	0.35 and 0.65
Air content, a_c_ [%]	6	4 and 8
Aggregate-to-cement (a/c) ratio	4.5	3 and 6
Curing duration, t_cu_ [days]	14	7 and 28
RH during curing, RH_cu_ [%]	75	50 and 100
Temperature during curing, T_cu_ [K]	298	283 and 313
Exposure duration, t_exp_ [years]	10	5 and 15
RH during exposure, RH_exp_ [%]	75	50 and 100
Temperature during exposure, T_exp_ [K]	298	283 and 313
Diffusion coefficient in capillary water at 298 K, D_cw_ [m^2^/s]	2.03 × 10^−9^	2.26 × 10^−9^ and 1.36 × 10^−9^
Critical RH, RH_c_ [%]	75	60 and 90
Activation energy for hydration reactions, ΔU_hyd_ [kJ/mol]	22.44	16.83 and 28.05
Activation energy for Cl^−^ diffusion in capillary water, Δ$U_{{\mathrm{Cl}}_{Diff}}$ [kJ/mol]	17.0	21.25 and 12.75
Activation energy for RH diffusion, Δ$U_{{\mathrm{RH}}_{Diff}}$ [kJ/mol]	38.9	29.125 and 48.625
Exposure zone for surface Cl^−^ concentration, C_s_ (t) [%weight of concrete]	Spray zone $\left(0.24\times t_{\exp}^{0.47}\right)$	Tidal zone $\left(0.38\times t_{\exp}^{0.37}\right)$ Atmospheric zone $\left(0.12\times t_{\exp}^{0.54}\right)$

**Table 4:** Parameters used for validation study.

Parameters	Andrade_04	Andrade_06	Sergi_05
Cement type	Type I	Type I	Type I
Aggregate type	Dense crushed limestone	Dense crushed limestone	N.A.
Tri-calcium silicate (C_3_S) [%]	59.8 *	59.8 *	41.3
Di-calcium silicate (C_2_S) [%]	20.7 *	20.7 *	27
Tri-calcium aluminate (C_3_A) [%]	8.1 *	8.1 *	14.2
Tetra-calcium aluminoferrite (C_4_AF) [%]	11.5 *	11.5 *	16.1
Water-cement (w/c) ratio	0.4	0.6	0.5
Air content, a_c_ [%]	7.9	8	10 *
Aggregate-to-cement (a/c) ratio	1.9 *	1.9*	0
Curing duration, t_cu_ [days],	14	14	90
RH during curing, RH_cu_ [%]	100	100	100
Temperature during curing, T_cu_ [K]	296	296	293
Age of concrete at the start of the exposure [days]	104	104	90
Exposure duration, t_exp_ [days]	90	90	100
RH during exposure, RH_exp_ [%]	100	100	100
Temperature during exposure, T_exp_ [K]	296	296	298
Surface Cl^−^ concentration at the end of the exposure period, C_s_ [M]	0.75	0.552	1.00
Temporal function of surface Cl^−^ concentration, C_s_ (t) [M]*	C_s_ × log((10 × t/t_exp_ + 1)/1.1)	C_s_ × log((10 × t/t_exp_ + 1)/1.1)	C_s_ × log((10 × t/t_exp_ + 1)/1.1)
Cl^−^ diffusion coefficient in capillary water at 298 K, D_cw_ [m^2^/s] *	2.03 × 10^−9^	2.03 × 10^−9^	2.03 × 10^−9^
Critical RH, RH_c_ [%] *	75	75	75
Activation energy for hydration reactions, ΔU_hyd_ [kJ/mol] *	22.44	22.44	22.44
Activation energy for Cl^−^ diffusion in capillary water, Δ$U_{{\mathrm{Cl}}_{Diff}}$ [kJ/mol] *	17.0	17.0	17.0
Activation energy for RH diffusion, Δ$U_{{\mathrm{RH}}_{Diff}}$ [kJ/mol] *	38.9	38.9	38.9

N.A.: Not applicable. * Not provided in the references and assumed for modeling purposes based on best estimates.

**Table 5:** Modeling parameters used in the case study.

Parameters	Values
Cement type	Type I
Aggregate type	Dense crushed limestone
Tri-calcium silicate (C_3_S) [%]	60.44
Di-calcium silicate (C_2_S) [%]	20.88
Tri-calcium aluminate (C_3_A) [%]	10.99
Tetra-calcium aluminoferrite (C_4_AF) [%]	7.69
Water-cement (w/c) ratio	0.50
Air content, a_c_ [%]	6
Aggregate-to-cement (a/c) ratio	4.5
Curing duration, t_cu_ [days]	14
RH during curing, RH_cu_ [%]	See Figure 10
Temperature during curing, T_cu_ [K]	See Figure 10
Age of concrete at the start of the exposure [days]	14
Exposure duration, t_exp_ [years]	15
RH during exposure, RH_exp_ [%]	See Figure 10
Temperature during exposure, T_exp_ [K]	See Figure 10
Temporal function of surface Cl^−^ concentration, C_s_ (t) [M]	See Equation (32)
Temporal function of surface O_2_ concentration, O_2_ (t) [M]	3 × 10^−4^
Cl^−^ diffusion coefficient in capillary water at 298 K, D_cw_ [m^2^/s]	2.03 × 10^−9^
Critical RH, RH_c_ [%]	75
Activation energy for hydration reactions, ΔU_hyd_ [kJ/mol]	22.44
Activation energy for Cl^−^ diffusion in capillary water, Δ$U_{{\mathrm{Cl}}_{Diff}}$ [kJ/mol]	17.0
Activation energy for RH diffusion, Δ$U_{{\mathrm{RH}}_{Diff}}$ [kJ/mol]	38.9

## Data Availability

The data presented in this study are available upon reasonable request from the corresponding author.
